# Swinepox virus vector-based vaccines: attenuation and biosafety assessments following subcutaneous prick inoculation

**DOI:** 10.1186/s13567-018-0510-5

**Published:** 2018-02-07

**Authors:** Xiaomin Yuan, Huixing Lin, Bin Li, Kongwang He, Hongjie Fan

**Affiliations:** 1 MOE Joint International Research Laboratory of Animal Health and Food Safety, College of Veterinary Medicine, Nanjing Agricultural University, Nanjing, China; 2 College of Veterinary Sciences, Fujian Agricultural and Forestry University, Fuzhou, China; 3 Institute of Veterinary Research, Jiangsu Academy of Agricultural Sciences, Nanjing, China; 4 Jiangsu Co-innovation Center for Prevention and Control of Important Animal Infectious Diseases and Zoonoses, Yangzhou, China

## Abstract

Swinepox virus (SPV) has several advantages as a potential clinical vector for a live vector vaccine. In this study, to obtain a safer and more efficient SPV vector, three SPV mutants, Δ003, Δ010, and ΔTK were successfully constructed. A virus replication experiment showed that these SPV mutants had lower replication abilities compared to wtSPV in 10 different host-derived cell lines. Animal experiments with mouse and rabbit models demonstrate that these three mutants and wtSPV did not cause any clinical signs of dermatitis. No fatalities were observed during a peritoneal challenge assay with these mutants and wtSPV in a mouse model. Additionally, the three mutants and wtSPV were not infectious at 60 h after vaccination in rabbit models. Furthermore, we evaluated biosafety, immunogenicity and effectiveness of the three mutants in 65 1-month-old piglets. The results show that there were no clinical signs of dermatitis in the Δ003 and ΔTK vaccination groups. However, mild signs were observed in the Δ010 vaccination groups when virus titres were high, and apparent clinical signs were observed at the sites of inoculation. Samples from all experimental pig groups were assessed by qPCR, and no SPV genomic DNA was found in five organs, faeces or blood. This suggests that the infectious abilities of wtSPV and the SPV mutants were poor and limited. In summary, this study indicates that two mutants of SPV, Δ003 and ΔTK, may be promising candidates for an attenuated viral vector in veterinary medicine.

## Introduction

Swinepox virus is a dsDNA virus that naturally infects only swine and is a member of the poxvirus family. With the advent of recombinant DNA techniques in the 1980s, Swinepox virus (SPV) was first considered as a recombinant vaccine vector almost 20 years ago [1, 2]. Subsequently, recombinant SPV (rSPV) was tested as a vaccine delivery vector for various swine infectious diseases, most notably *Streptococcus suis* and swine influenza, because it is non-pathogenic and possesses the ability to carry and express large amounts of foreign genetic material [3–8].

Given the interest in developing more effective rSPV vaccines and expression vectors, we sequenced and analysed the genome of a pathogenic SPV. SPV genome sequences were assembled into a contiguous sequence of approximately 146 kb, as in a previous report [9]. SPV has a compact gene arrangement with almost no overlapping ORF and no evidence of introns or large regions of noncoding DNA. SPV contains 150 putative genes that encode proteins of 53–1959 amino acids, of which 146 are poxvirus homologues [10]. Many swinepox virus gene functions are unknown. However, functions may be inferred based on the functions of other chordo poxviruses (ChPV). Since the first demonstration in 1982 of the ability of vaccinia virus to express an inserted herpes simplex virus thymidine kinase (TK) gene [11], a large variety of foreign genes have been expressed by recombinant vaccinia viruses. To date, *Streptococcus suis*, porcine circovirus, swine influenza virus, swine infectious gastroenteritis virus and other pathogenic protective antigen genes have been successfully expressed in swinepox virus [3–8]. Swinepox virus has broad application potential as a live vector of the swine vaccine.

Various delivery routes employed for viral vaccine vectors induce a diverse range of host immune responses to vaccine antigens [12–14]. *Plasmodium berghei malaria* and *Mycobacterium tuberculosis* antigens inserted into rFPV/rMVA and administered in a prime-boost regimen induce strong antigen-specific T cell responses [14, 15]. Furthermore, the co-expression of certain chemokines and cytokines has been demonstrated to enhance host immune responses to vaccine antigens [16–23]. These studies showed that rational combinations of vectors, delivery routes and co-stimulatory molecules play crucial roles in regulating immune responses to vaccine antigens.

For live viral vectors such as vaccina vectors, biosafety assessments have been deemed essential before clinical applications. A large number of studies have sought to reduce the risk of the vector itself to avoid adverse reactions [24]. Modified vaccinia virus Ankara (MVA) was one of the first reported VACV strains shown to be highly attenuated in animal models and safe in human trials [25, 26]. MVA was isolated in the 1960s from the chorioallantoic vaccinia virus Ankara strain (CVA) via extensive consecutive passaging in chicken embryo fibroblasts, a process that resulted in six major deletions within 122 of the 195 open reading frames (ORF) [27–29]. The best property of MVA is its failure to multiply in the vast majority of mammalian cells, particularly human cells. Three strategies have been commonly used to investigate the attenuation and biosafety of pox virus strains. (i) A novel attenuated virus vector constructed by gene engineering. The current method for VACV modification is based on homologous recombination in mammalian cells [30]. (ii) An attenuated virus obtained using routine methods. A viral vector is extensively serially passaged in established cell lines, resulting in the mutation of major pathogenesis-associated genes and the creation of a milder strain [27–29]. (iii) A novel virus strain newly found in nature. Some novel strains are naturally mild and attenuated, and these strains may be vaccine vector candidates.

Due to the development of gene editing technology, attenuated viruses obtained by genetic engineering are a hot spot in vaccine research. These vectors not only exert stronger immune effects and demonstrate higher safety but also cost less than those obtained by conventional methods. The use of swinepox virus as a viral vaccine vector has drawn widespread attention, but only a few studies have reported the optimization of swinepox virus attenuation. In a previous study, our group isolated and identified a novel strain of swinepox virus. The newly isolated SPV strain displayed mild and attenuated characteristics, and it was not fatal to either the host or non-host animals. However, swinepox virus also possesses a certain degree of virulence. We expect to perform biosafety assessments on this newly isolated attenuated SPV strain before it can be used in the veterinary clinic.

We have shown that rSPV is an excellent novel vaccine vector compared to traditional vaccines and that vaccine-vector combinations generate vastly different immune outcomes in a prime-boost setting. In particular, SPV has been considered a high biosafety standard live vaccine vector [3–6, 8]. These studies have further confirmed that SPV can be optimized to become a veterinary live vector with high biosafety.

## Materials and methods

### Facility and ethics statements

All experiments were conducted in the Key Laboratory of Animal Bacteriology, Ministry of Agricultural, Education Department of the College of Veterinary Medicine, Nanjing Agricultural University. Animal studies were approved by the Laboratory Animal Monitoring Committee of Jiangsu Province and by the Ministry of Agriculture of the College of Veterinary Medicine, Nanjing Agricultural University. The study was performed in strict accordance with the recommendations in the Guide for the Care and Use of Laboratory Animals. Protocols for the animal studies were approved by the Committee on the Ethics of Animal Experiments of the College of Veterinary Medicine, Nanjing Agricultural University (No. SYXK2016-0006).

### Virus strains, cells and medium

Wild-type swinepox virus (wtSPV [NT1501 strain]) was newly isolated and stored in our laboratory. The following cells were stored in our laboratory: Vero cells, African green monkey kidney cells; PK-15, porcine kidney cells; DF-1, chicken fibroblast cells: LLC, Lewis lung cancer cells; BHK-21, baby hamster Syrian kidney cells; MDCK, Madin-Darby canine kidney cells; MDBK, Madin-Darby bovine kidney cells; RK-13, rabbit kidney cells; F81, feline kidney cell cells; and HeLa cells. These cells were cultured in Dulbecco’s modified essential medium (DMEM), and all cells were maintained at 37 °C in a 5% (v/v) CO_2_ atmosphere.

### Construction of the deletion mutant vector

**Figure 1:**
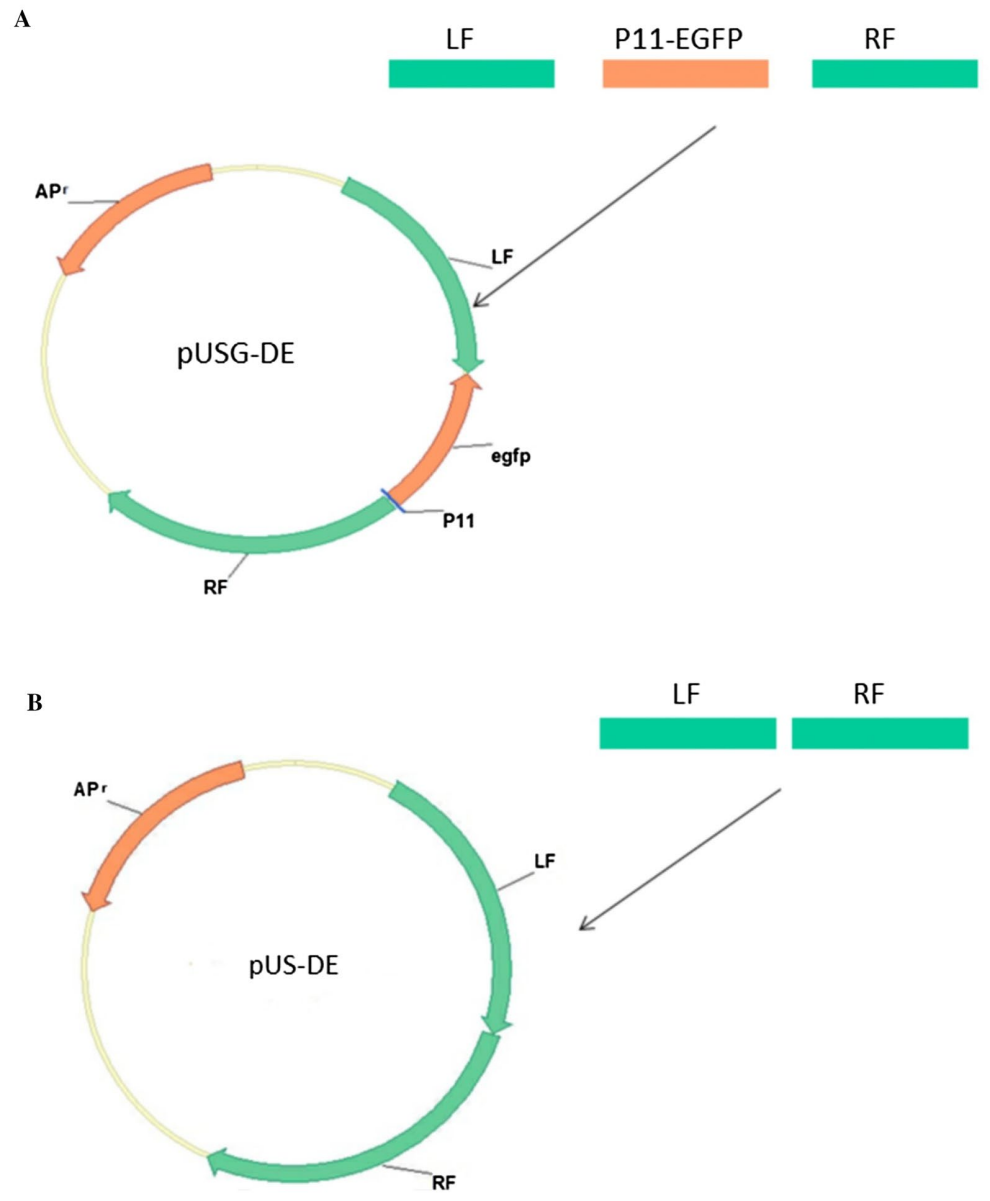
**Vector construction diagram.** **A** pUSG-DE; **B** pUS-DE. LF and RF indicate the left flanking sequences and right flanking sequences, respectively, of SPV. P11 is a vaccinia virus (VV) promoters. The GFP reporter gene is also included in the plasmid.

The primers used in this study are listed in Table 1. Using the swine pox virus genome as a template, primers L1/L2 and R1/R2 amplified the corresponding 003, 010, TK gene LF and RF. The synthetic plasmid pEGFP N1-Pdx1 PCR primer was used as a template with EGFP-P11-1 (5′-GCGGCCGCTTTACTTGTACA-3′) and EGFP-P11-2 (5′-GTCGAGATATAGTAGAATTTCATTTTGTTTTTTTCTATGCTATAAATGAA-3′). The fragment contains the SPV specific promoter P11. The overlapping PCR technique was used to generate targeted constructs for LF, EGFP-P11 and RF (pUS-DE vector containing only LF ad RF). PCR amplified products were purified from a 1% agarose gel using Gel PCR DNA Fragments Extraction Kit (Geneaid, Taiwan) and then ligated into the pMD-19T vector. The resulting vectors were designated pUSG-003/010/TK and pUS-003/010/TK (Figure 1).

**Table 1:** **SPV deletion vector pUSG-DE and pUS-DE to construct primers**

Vector	Target	Primer	Primers for plasmid construction (5′–3′)
pUSG-DE	003	003LF-forward-1 003LF-reverse-1 003RF-forward-1 003RF-reverse-1	5′ CCAATAGGATGCGGCTCT 3′ 5′ TGTACAAGTAAAGCGGCCGCAGCTTTTCACTTTTTGATGT 3′ 5′ AAATTCTACTATATCTCGACTATGATTTATTTTTGGAATA 3′ 5′ TCCTATAAAGCGACAACC 3′
	010	010LF-forward-1 010LF-reverse-1 010RF-forward-1 010RF-reverse-1	5′ ATCTCACATACTCTGCCA 3′ 5′ TGTACAAGTAAAGCGGCCGCTTTTTTTATCAAAACTGAAG 3′ 5′ AAATTCTACTATATCTCGACTATTGTTATTTATTTGAATA 3′ 5′ ATATCCTATAATGGAAGAAG 3′
	TK	TKLF-forward-1 TKLF-reverse-1 TKRF-forward-1 TKRF-reverse-1	5′ TTGCTTTAGCTGGTAAGT 3′ 5′ TGTACAAGTAAAGCGGCCGCAAAGTGTTTATTTATTTTTC 3′ 5′ AAATTCTACTATATCTCGACAATATTGAAAATATAATTAA 3′ F2 5′ ATAGTCATGTTTGCGACCAT 3′
pUS-DE	003	003LF-forward-2 003LF-reverse-2 003RF-forward-2 003RF-reverse-2	5′ CCAATAGGATGCGGCTCT 3′ 5′ AGCTTTTCACTTTTTGATGT 3′ 5′ ACATCAAAAAGTGAAAAGCTTATGATTTATTTTTGGAATA 3′ 5′ TCCTATAAAGCGACAACC 3′
	010	010LF-forward-2 010LF-reverse-2 010RF-forward-2 010RF-reverse-2	5′ ATCTCACATACTCTGCCA 3′ 5′ TTTTTTTATCAAAACTGAAG 3′ 5′ CTTCAGTTTTGATAAAAAAATATTGTTATTTATTTGAATA 3′ 5′ ATATCCTATAATGGAAGAAG 3′
	TK	TKLF-forward-2 TKLF-reverse-2 TKRF-forward-2 TKRF-reverse-2	5′ TTGCTTTAGCTGGTAAGT 3′ 5′ AAAGTGTTTATTTATTTTTC 3′ 5′ GAAAAATAAATAAACACTTTAATATTGAAAATATAATTAA 3′ 5′ ATAGTCATGTTTGCGACCAT 3′

### Generation and screening of swinepox virus deletion mutants

A sub-confluent culture of PK-15 cells was infected with wtSPV (0.02 multiplicity of infection [MOI]) for 2 h. Then, the cells were transfected with 10 µg of the pSPVG-003, pSPVG-010 or pSPVG-TK plasmid using Exfect™ Transfection Reagent (Vazyme Biotech Co., Ltd). After 72 h in culture, the PK-15 cells were harvested and lysed via five rounds of freezing and thawing. The lysate was then used to infect PK-15 cells in a 12-well plate to plaque-purify virus deletion mutants. Briefly, for plaque purification with fluorescence microscopy (Zeiss, Germany), 1.5 mL of medium containing 1% LMP agarose (DingGuo, Beijing, China) was added to each well of the 12-well plate, and the infected cells were incubated for 5 days until plaques became visible under a light microscope. The plaques were resuspended in 0.3 mL of medium containing 2% foetal bovine serum. Plaque purification was repeated for 3–5 rounds until all plaques in a given well showed green fluorescence. Deletion strains expressing green fluorescent protein were designated Δ003-G, Δ010-G and ΔTK-G.

The deletion viruses labelled with green fluorescence (Δ003-G, Δ010-G and ΔTK-G) were then re-plated into a 6-well culture plate containing PK-15 cells and the second transfection was performed with pUS-003, pUS-010 and pUS-TK. The culture medium was collected on the fifth day after transfection and then incubated twice with new PK-15 cells in a 12-well plate. After incubation at 37 °C for 2 h, medium was added to the semi-solid medium containing 1% methylcellulose of DMEM. The plaques were resuspended in 0.3 mL of medium containing 2% FBS. Plaque purification was repeated for 3–5 rounds using an ordinary microscope and a fluorescence microscope, therefore screening the deletion mutant without GFP. The obtained virus strains were the traceless deletion strains Δ003, Δ010 and ΔTK.

### Virus purification and concentration

SPV was propagated in PK-15 cells and purified using a sucrose density gradient separation. Briefly, the virus was released by three rounds of freezing and thawing of the cell cultures, and then was concentrated from the supernatant via ultracentrifugation at 120 000 × *g* for 1.5 h at 4 °C. The viral pellet was then resuspended in phosphate buffered saline (PBS), layered on 30 and 60% (w/v) sucrose gradients, and centrifuged at 100 000 × *g* for 2 h and 4 °C. The purified virus band was collected and resuspended in PBS, and centrifuged at 120 000 × *g* for 1.5 h at 4 °C to pellet the purified virus. Finally, the purified-SPV was resuspended in PBS and stored at −80 °C.

### Growth characterization of SPV deletion mutants in vitro

To analyse the growth characteristics of SPV virus in vitro, a growth curve was constructed in PK-15 cells. Confluent PK-15 cells seeded in 24-well plates were infected with SPV deletion mutants and wtSPV at the same multiplicity of infection (MOI) of 0.1. The infected PK-15 cells were harvested at 6, 12, 24, 36, 48, 60, 72 and 84 post-infection. The titres of viruses harvested at different time points were determined by performing a virus plaque experiment and calculating plaque forming units (PFU). All in vitro experiments were performed in triplicate.

### Virus replication characteristics in cells

The replication capacity and genetic stability of deletion mutants were evaluated using ten different cell lines. PFU calculation was performed using an evaluation method for deletion mutants and wild SPV replication, yielding differences in different cells. The subculture of viruses in different cell lines was performed to evaluate their genetic stability.

Viral loads in cell samples were detected for virus-infected PK-15 cells as previously described [31–33]. Confluent PK-15 cell monolayers in 12-well cell culture plates were inoculated with virus at a multiplicity of infection (MOI) of 0.01. Δ003, Δ010, ΔTK or wtSPV infected cells were collected at different time points (0, 12, 24, 36, 48, 60, and 72 h). The supernatants were collected to extract viral DNA with a MiniBEST Viral RNA/DNA Extraction Kit ver. 5.0 (Takara). A pair of SPV genome-specific primers (5′-AAACATCATCATCGCTAC-3′/5′-TACCAGGAGAATGAAAAG-3′) was designed. A 100-bp gene fragment was amplified by PCR from SPV genomic DNA using these two primers and inserted into the Simple 19T vector (Takara) to construct the 19T-V plasmid. After confirmation, 19T-V was transformed into competent *Escherichia coli* DH5α to prepare large amounts of 19T-V plasmid DNA to establish a standard curve for real-time PCR.

### Virulence in mice

To determine the safety of these viral vectors in mice, we evaluated six virus titres (1 × 10^1^, 1 × 10^2^, 1 × 10^3^, 1 × 10^5^, 1 × 10^6^ and 1 × 10^7^ PFU/mL). (i) Four groups of 4-week-old mice (ICR, BALB/C and nude) were used as animal models. Ten randomly selected mice from each group were infected with Δ003, Δ010, ΔTK or wtSPV, via intradermal injection (100 μL of viral medium, per os), and observed for 30 days. (ii) Then, 2-week-old and 6-week-old BAB/C female mice were subjected to abdominal challenge (100 μL viral medium, 1 × 10^3^ PFU/mL). Each group of 10 was divided into four groups (Δ003, Δ010, ΔTK or wtSPV) and observed for 30 days. (iii) Forty new-born BALB/C mice were divided into 4 groups (Δ003, Δ010, ΔTK or wtSPV) of 10. Vaccine solution was orally administered (50 μL viral medium) via oral gavage using a 5-mL syringe; the sharp syringe head was removed to insert the syringe into the mouth of the animal, slowly pushing the liquid in the syringe into the animal’s oesophagus.

### Evaluation of viral virulence in rabbits

To determine the safety of these viral vectors in mice, we evaluated six virus titre doses (1 × 10^1^, 1 × 10^2^, 1 × 10^3^, 1 × 10^5^, 1 × 10^6^ and 1 × 10^7^ PFU/mL). Four groups of 6-month-old New Zealand rabbits were used as animal models. Five randomly selected rabbits from each group were infected with Δ003, Δ010, ΔTK or wtSPV, via intradermal injection (1 mL of viral medium, per os).

Another group of rabbits was used to assess virus clearance. Each of three rabbits was inoculated intradermally at multiple sites with 0.1 mL of PBS containing 10^3^ PFU/mL of each test virus or PBS alone. Indurations and ulcerations were measured and recorded. After 60 h, the rabbits were euthanised, and skin biopsy specimens collected from each of the inoculation sites were aseptically prepared via mechanical disruption and indirect sonication for virus recovery. Infectious viruses were assayed by plaque titration on CEF monolayers.

### Evaluation of lesions in swine

To test the efficacy of the SPV deletion in piglets, sixty-one-month-old piglets (verified as SPV negative) were randomly divided into four groups (*n* = 15/group, five one-month-old piglets as the blank control group). The four virus groups were divided into three dilutions (10^3^, 10^2^ and 10^1^ PFU/mL) and were skin pricked for inoculation as follows: (1) Δ003; (2) Δ010; (3) ΔTK; and (4) wtSPV. Five pigs were inoculated with PBS as negative controls. The inoculation sites were in the right front legs. After the first inoculation, we collected the serum, faeces, mosquitoes, and water source within one kilometre of the detected pathogenic microorganisms every 3 days. All pigs were observed daily for skin and mental state and for general signs of disease. The mean weight change was calculated from the body weights of five pigs recorded daily after infection with 10^3^ PFU/mL relative to normally growing pigs. All observations described in this study were conducted by five people in our laboratory in a blinded and randomised manner.

### Indirect ELISA to determine antibody titres

Serum was collected at 0, 7, 14, 21, 28, 35, 42 and 49 days post-primary immunisation from the vaccinated pigs, and an indirect ELISA was used to detect SPV-specific antibodies. We used purified SPV in 100 μL of PBS (pH 7.2) at an empirically optimised dilution to coat 96-well plates. The virus was added to the plates and then incubated overnight at 4 °C. The following morning, the plates were washed three times with PBST and then blocked with 5% skim milk (in PBST) at 37 °C for 2 h. Serum samples were serially diluted and incubated at 37 °C for 1 h. The samples were set up at the same time and divided into the following five groups: (1) Δ003 positive serum, (2) Δ010 serum, (3) ΔTK serum, (4) wtSPV positive serum, and (5) blank control. After incubation, the plates were washed three times with PBST, and then horseradish peroxidase (HRP)-conjugated goat anti-SPA IgG (1:10 000 diluted in PBST, Signalway Antibody) was added to each well. The plates were incubated at room temperature in the dark for 30 min and then washed three times with PBST. The TMB microwell peroxidase substrate system (TIANGEN) was used to develop the reaction. Samples were developed for 20 min, and the reaction was stopped with 2.0 M sulfuric acid. All assays were performed in duplicate. A micro plate reader (Bio-Rad) was used to measure the reaction products at an absorbance of 450 nm [34].

### Quantitation of viral loads

Quantitative assays were carried out to measure viral loads in the blood and faeces as previous described [31, 33]. Serum and faeces were collected from all experimental pigs at 0, 7, 14, 21, 28, 35, 42 and 49 days post-inoculation. Tissue samples from five organs (heart, liver, spleen, lung, and kidney) were collected from pigs at 28 and 49 days. SPV genomic copies were detected by real-time PCR. Serum, faeces and tissue samples were homogenised in PBS at a ratio of 1:5 (g/mL) and centrifuged at 10 000 × *g* for 30 min. The supernatants were collected to extract viral DNA with a MiniBEST Viral RNA/DNA Extraction Kit ver. 5.0 (Takara, Japan). The sensitivity and specificity of the real-time PCR assay were then determined, referring to the viral load test of the cells above.

### Cytokine assay

A cytokine assay was performed as described previously [35]. The cellular immune response type was indirectly assessed by measuring IFN-γ and IL-4 levels in the serum. The pigs were sacrificed at 30 days after the first immunisation with wtSPV, Δ003, Δ010, ΔTK or PBS. Cytokines were detected using ELISA kits (ExCell Bio, China) according to the manufacturer’s instructions. Standard curves were generated using control IFN-γ and IL-4 serially diluted twofold in PBS and coated onto ELISA plates overnight at 37 °C. Serum IFN-γ and IL-4 were calculated according to their corresponding standard curves.

### Neutralising antibody assay

Assays to detect neutralising antibodies were performed as described previously [6, 7]. Serum was collected at 0, 7, 14, 21, 35, and 42 days post-primary immunisation from pigs immunised with wtSPV, Δ003, Δ010, ΔTK or PBS. The serum was diluted from 1:4–1:1024 in a 100-μL volume. The sera were mixed with an equal volume of 70 PFU/mL SPV at 37 °C. After incubation for 1.5 h, the antibody-virus mixtures were used to infect PK-15 cells in 96-well plates, and the cells were overlaid with agar. The cells were grown for 3 days at 37 °C in a 5% CO2 atmosphere to detect SPV-specific CPE.

### Statistical analysis

All data were analysed using one-way ANOVA. The threshold for significance was *P* < 0.05.

## Results

### Characterisation of SPV deletion mutants

**Figure 2:**
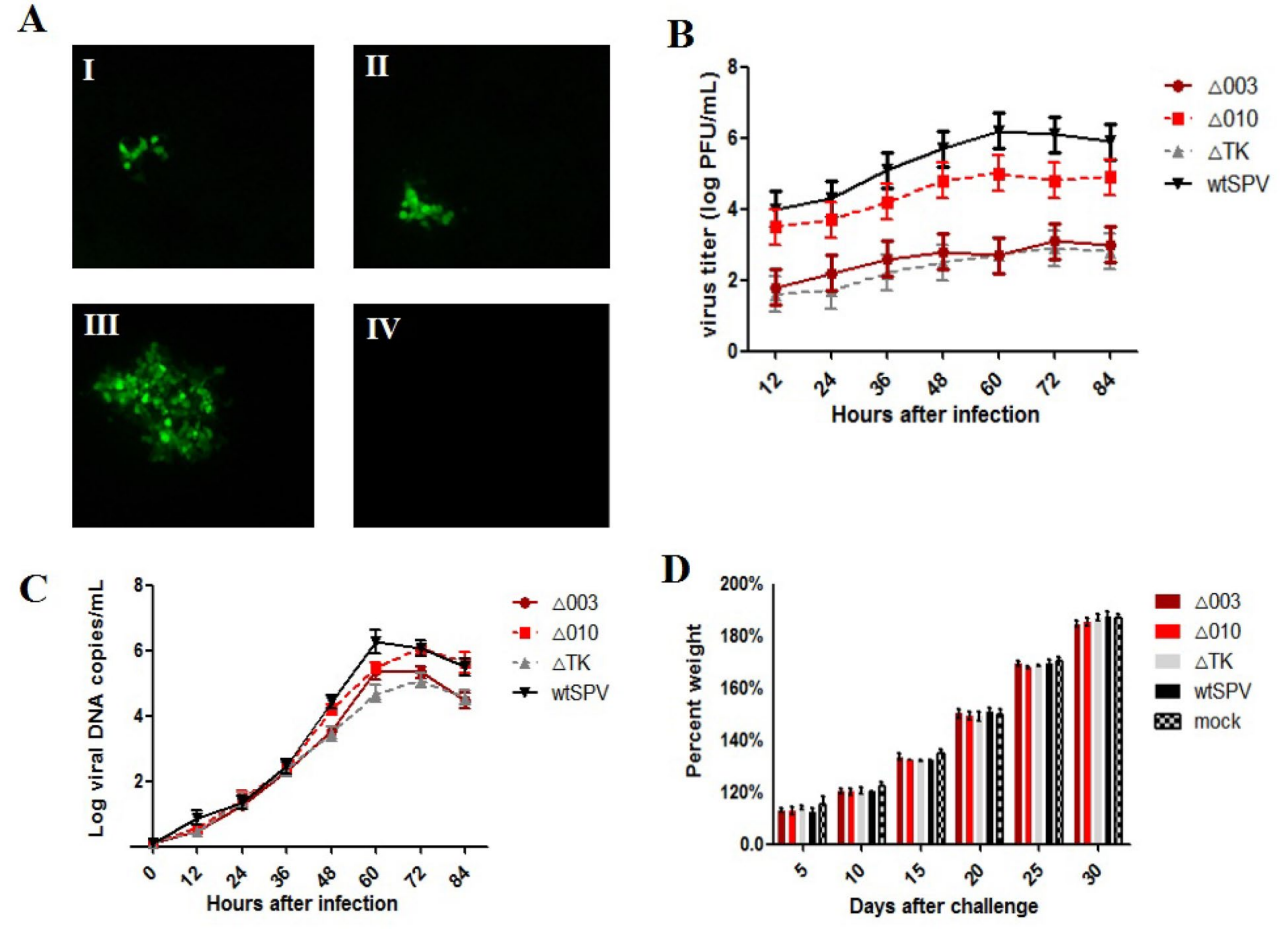
**The virus replication characteristics in cells and mice. A** The swine pox virus deletion strain was observed under a fluorescence microscope at 72 h post-infection (I) Δ003-G, (II) ΔTK-G, (III) Δ010-G, and (IV) PK-15 cells. Magnification 100×. **B** Growth kinetics curves of Δ003, Δ010, ΔTK and wtSPV in PK-15 cell cultures. **C** Measurement of viral loads in PK-15 cells by qPCR. **D** Changes in body weight of BALB/c mice after inoculation with Δ003, Δ010, ΔTK and wtSPV. The percent body weight change was calculated from the body weights of 10 mice recorded daily after infection relative to that at day 0 (pre-infection). *P < 0.05.

To confirm the construction of SPV deletion mutants, PCR was used to amplify the deletion genes. The Δ003, Δ010, and ΔTK mutants contained 1020, 258, and 543 bp fragment deletions, respectively. The titre of the Δ010 mutant was approximately 1 × 10^5^ PFU/mL. The titres of the Δ003 and ΔTK mutants were lower than those of the Δ010 mutant and wtSPV, at approximately 1 × 10^3^ PFU/mL. Plaque assays were used to confirm the presence of deletion mutants, as indicated by green fluorescence (Figure 2A). The growth characteristics of deletion mutants and wtSPV are shown in Figure 2B. After the deletion mutants and wild type virus were purified and concentrated, the following virus titres were determined in PK-15 cells: 1 × 10^7.2^ PFU/mL (Δ003), 1 × 10^7.4^ PFU/mL (Δ010), 1 × 10^7.1^ PFU/mL (ΔTK) and 1 × 10^8.1^ PFU/mL (wtSPV).

### Characterisation in cells

Following evaluation in porcine cell lines and non-porcine-derived cell lines, we determined that the titres of wtSPV and deletion mutant viruses were higher in PK-15 and Vero cells than in non-porcine cell lines. In the majority of non-swine cells, virus replication did not survive passage for more than three generations. Virus titres in the experimental group cells were lower than those in the wild-type SPV and the Δ010 groups in the non-porcine cell line but higher than those in the Δ003 and ΔTK groups. The ΔTK group titres were minimal (Table 2).

**Table 2:** **Virus replication of Δ003, Δ010, ΔTK, and wtSPV in different cell lines (log PFU/mL)**

	Cell line	N1	N2	N3	N4	N5	N6	N7	N8	N9	N10
Δ003	PK-15	2.8	2.9	3.0	2.9	3.0	3.1	2.9	3.0	2.9	2.9
	Vero	3.2	3.2	3.0	3.2	3.2	3.2	3.2	3.0	3.2	3.2
	DF-1	2.7	1.7	1.5	ND	ND	ND	ND	ND	ND	ND
	LLC	1.2	ND	ND	ND	ND	ND	ND	ND	ND	ND
	BHK	1.2	ND	ND	ND	ND	ND	ND	ND	ND	ND
	MDCK	1.0	ND	ND	ND	ND	ND	ND	ND	ND	ND
	MDBK	1.0	ND	ND	ND	ND	ND	ND	ND	ND	ND
	F81	1.2	ND	ND	ND	ND	ND	ND	ND	ND	ND
	RK13	2.7	2.0	2.0	ND	ND	ND	ND	ND	ND	ND
	HELA	2.7	2.0	2.0	ND	ND	ND	ND	ND	ND	ND
Δ010	PK-15	5.0	4.9	4.8	4.9	5.0	5.0	5.0	4.8	4.8	4.9
	Vero	5.2	5.2	5.0	5.2	5.2	5.0	5.1	5.2	5.2	5.2
	DF-1	3.5	2.6	1.7	ND	ND	ND	ND	ND	ND	ND
	LLC	1.4	ND	ND	ND	ND	ND	ND	ND	ND	ND
	BHK	1.2	ND	ND	ND	ND	ND	ND	ND	ND	ND
	MDCK	1.0	ND	ND	ND	ND	ND	ND	ND	ND	ND
	MDBK	1.0	ND	ND	ND	ND	ND	ND	ND	ND	ND
	F81	1.2	ND	ND	ND	ND	ND	ND	ND	ND	ND
	RK13	3.7	3.0	2.0	ND	ND	ND	ND	ND	ND	ND
	HELA	4.7	3.8	2.0	ND	ND	ND	ND	ND	ND	ND
ΔTK	PK-15	2.8	2.9	2.8	2.8	2.8	2.8	2.8	2.8	2.8	2.8
	Vero	3.1	3.2	3.0	3.2	3.2	3.0	3.0	3.0	3.0	3.0
	DF-1	2.7	1.7	1.2	ND	ND	ND	ND	ND	ND	ND
	LLC	1.4	ND	ND	ND	ND	ND	ND	ND	ND	ND
	BHK	1.1	ND	ND	ND	ND	ND	ND	ND	ND	ND
	MDCK	1.0	ND	ND	ND	ND	ND	ND	ND	ND	ND
	MDBK	1.0	ND	ND	ND	ND	ND	ND	ND	ND	ND
	F81	1.2	ND	ND	ND	ND	ND	ND	ND	ND	ND
	RK13	2.6	2.2	1.8	ND	ND	ND	ND	ND	ND	ND
	HELA	2.8	2.0	1.6	ND	ND	ND	ND	ND	ND	ND
wtSPV	PK-15	6.1	5.9	6.0	6.2	6.0	5.9	5.9	6.0	6.0	6.1
	Vero	5.2	5.2	5.0	5.2	5.2	5.2	5.0	5.2	5.2	5.2
	DF-1	3.7	2.8	1.8	ND	ND	ND	ND	ND	ND	ND
	LLC	1.4	ND	ND	ND	ND	ND	ND	ND	ND	ND
	BHK	1.2	ND	ND	ND	ND	ND	ND	ND	ND	ND
	MDCK	1.0	ND	ND	ND	ND	ND	ND	ND	ND	ND
	MDBK	1.0	ND	ND	ND	ND	ND	ND	ND	ND	ND
	F81	1.2	ND	ND	ND	ND	ND	ND	ND	ND	ND
	RK13	3.7	2.5	2.0	ND	ND	ND	ND	ND	ND	ND
	HELA	4.7	3.0	2.2	ND	ND	ND	ND	ND	ND	ND

ND: not detected.

### Assessment of attenuation of the deletion mutants in mice

When we tested six virus titres via mouse inoculation, we obtained the following results: (i) the intradermal injection of different strains of SPV, Δ003, Δ010, ΔTK and wtSPV into 4-week-old mice did not lead to pox virus-related disorders in mice at either high or low doses of virus. Genomic copies of SPV were not detected in any serum and faeces samples after 15 days of challenge. Mice infected with Δ003, Δ010, ΔTK and wtSPV did not show weight loss (Figure 2D). (ii) Two-week-old and 6-week-old BALB/C mice were injected intraperitoneally. The results show that both the deletion mutants and wtSPV did not cause death. (iii) One-day-old BALB/C mice were orally administered deletion mutants and wtSPV and had no adverse reactions, indicating that the oral administration of swinepox virus is safe.

### Evaluation in rabbit models

Intramuscular injection showed that the injection of wtSPV and ∆010 at 10^7^ PFU/mL and 10^6^ PFU/mL into rabbits resulted in a 0.1-cm diameter pox rash and a slight increase in weight at 8 dpi, followed by recovery at 20 dpi. No adverse effects were observed in rabbits vaccinated with Δ003 and ΔTK individually via the intradermal route. The skin and tissue at the vaccine administration position were surgically removed and homogenised 60 h after vaccination, and applied to the PK-15 cell based plaque assay. No induration or ulcerative lesions were observed at the inoculation sites for Δ003, Δ010, ΔTK and wild type SPV. These results indicate that the viruses were present at undetectable levels 60 h after vaccination.

### Evaluation in pigs

**Figure 3:**
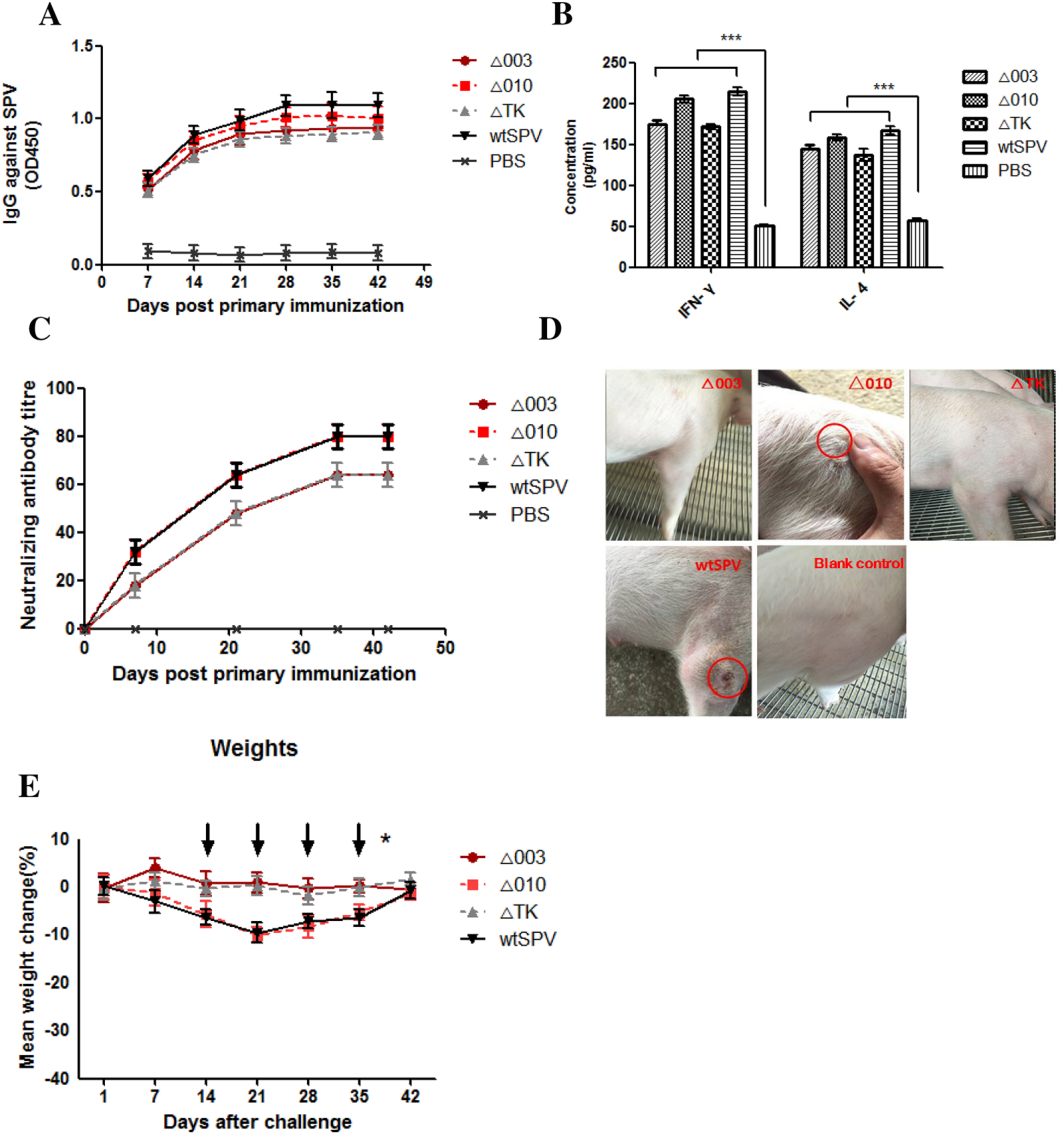
**Deletion and wtSPV testing in swine. A** Measuring the humoural immune response to the deletion mutants and wtSPV in pigs; **B** IL-4 and IFN-γ levels post-vaccination. The IL-4 and IFN-γ levels of the deletion mutant and wtSPV group at 30 days post-vaccination. **C** The deletion mutant and wtSPV groups induce SPV-specific neutralizing antibodies in pigs. The titres of neutralizing antibodies are expressed as the reciprocal of the highest serum dilution, in which no CPE was observed. **D** Photographic images of SPV deletion mutants and wtSPV at 10 days after inoculation. **E** Swine weight loss curves at 42 days post-inoculation. The data are shown as the mean ± S.D. Weight loss was analysed by two-way ANOVA (*P < 0.05; ***P < 0.001) for animals. Arrows indicate days on which the deletion mutants and wtSPV demonstrated statistically significant differences from the placebo vaccine.

At 5 days post-immunisation, pigs that received wtSPV had pox marks approximately 0.2 cm in diameter around the infection sites. At 10 days post-immunisation, the pox marks were significantly reduced (Figure 3D), and all pigs recovered after 15 days. High-virus-titre groups administered Δ010 also exhibited slight skin inflammation. As virus titres decreased, swinepox virus-related skin clinical signs were reduced. The deletion mutant dilutions administered to pigs resulted in no skin inflammation or other adverse reactions. The weight loss curves for pigs inoculated with a virus titre of 10^3^ PFU/mL are shown in Figure 3E. Indirect ELISA results for serum SPV antibody levels are shown in Figure 3A, and cytokine levels are shown in Figure 3B. Wild-type and deletion virus infections in experimental group pigs yielded undetectable levels of SPV virus. Neutralisation test results are shown in Figure 3C, 35 days after inoculation, wtSPV and Δ010 stimulated the host to produce neutralising antibody titres of 1:80, and Δ003 and ΔTK produced titres of 1:64. There were no indications of abnormal body temperature, appetite, or mental state in any of the vaccinated animals. On the 15th day after challenge, two pigs were randomly selected from each experimental group and sacrificed for biopsy. Internal organs, including heart, spleen, lung, kidney and intestine, were extracted and analysed for SPV viral load. Genomic copies of SPV were not detected in any serum and faeces samples at 0, 7, 14, 21, 28, 35, 42 and 49 days post-inoculation in any pigs.

An environmental release test showed that SPV was not present in mosquitoes, water sources and faeces collected every 3 days within a 1 km radius of the experimental pig. All pigs on the pig farms were SPV-negative by PCR at different time points.

## Discussion

Here, we engineered three gene knockout swinepox viruses, Δ003, Δ010 and ΔTK, and tested their replication in ten types of host cells, their virulence in ICR, BALB/C and nude mouse models, their induction of specific immune responses in New Zealand rabbits, and their vaccine efficacy in a pig model, and we also assessed their biosafety as potential live vectors. When the 003 and TK genes were knocked out, the replication capacity of these viruses in non-target animal cells decreased significantly (with the exception of Vero cells), and virus subculture did not exceed more than three generations. Neither wtSPV nor gene-deleted SPV infected non-target animals. The biosecurity of the three gene-deleted SPV was similar to that of the SPV, but the reduced virulence of these three mutant SPV was significant. In swine model experiments, the deletion mutants only caused dermatology related signs and did not spread to other organs. In the Δ003 and ΔTK challenge group, the inflammatory responses of inoculated pig skin completely disappeared, indicating that the safety of Δ003 and ΔTK was higher than that of wtSPV. However, a live vector itself is often immunogenic and affects the host immunity.

Neutralising antibody levels against these two deletion mutant SPV were decreased compared to that of wtSPV. Additionally, the TH1 and TH2 mediated immune response, e.g., interferon-γ and IL-4 levels, also decreased, the most highly significant decrease in these immune factors were observed for ΔTK-SPV among the three mutant deletion SPV. Intraspecific and interspecific transmission infection cases were not found in our study. A virus release experiment showed that the SPV deletion mutant did not cause large-scale virus spread, indicating that live viral vectors may be associated with notably low spreading. The TK gene of SPV is located in the middle of the viral genome, encoding a peptide that contains 175 amino acids. This SPV TK peptide shares 64% amino acid identity with the peptide encoded by the J2R gene of vaccina virus. An analysis showed that the homology between the pox virus TK gene and the herpes simplex virus TK gene was low. Even among TK genes from different pox viruses, the homology was also low. Nevertheless, there are several homologous regions. The TK gene encodes an enzyme that exists in both eukaryotes and pox viruses. This product phosphorylate thymidine riboside into mono-phosphoryl thymidine riboside, which is subsequently phosphorylated by an in vivo phosphatase into di- and tri-phosphoryl thymidine riboside, which then participates in DNA synthesis [36, 37]. Therefore, deletion of the TK gene would jeopardy the viral DNA replication, leading to decreased SPV virulence, and explains the dermatitis signs that appeared in wild type SPV inoculated pigs that were not observed in ΔTK SPV inoculated pigs.

The SPV 010 gene encodes the repressor of eIF2-like protein kinase R. PKR is an enzyme encoded by the EIF2AK2 gene that protects against viral infections. PKR is central to the cellular response to different stress signals, such as pathogens, lack of nutrients, cytokines, irradiation, mechanical stress, or ER stress. The PKR pathway leads to the stress response through the activation of other stress pathways, such as JNK, p38, NF-kB, PP2A and phosphorylation of eIF2α. ER stress caused by an excess of unfolded proteins leads to inflammatory responses. PKR contributes to this response by interacting with several inflammatory kinases, such as IKK, JNK, ElF2α, insulin receptor and others. This metabolically activated inflammatory complex is called the metabolic inflammasome or metaflammasome [38, 39]. After deletion of the SPV 010 gene, only a slight decrease in SPV virulence was detected; therefore, we propose that the SPV 010 gene may not be a key virulence gene.

The SPV 003 gene encodes the MHC class I alpha chain-like protein. The MHC class I proteins are a set of surface proteins in vertebrates and are involved in the endogenous antigen presenting process. Indeed, the ~9-amino-acid antigenic peptide generated from endogenous antigens bind to MHC class I, forming an antigenic peptide-MHC class I complex, which is subsequently presented to CD8+ cytotoxic T cells [40]. Here, we suggest that the MHC class I alpha chain-like protein encoded by the SPV 003 gene competitively inhibit the MHC class I protein-mediated antigen presenting process, intervening in the immune system and leading to immune-escape [10]. Therefore, deletion of the SPV 003 gene contributes to the significant decrease in SPV virulence and mild clinical dermatitis signs observed in our experiment, indicating that this gene is highly related to SPV virulence.

In this study, we show that (i) SPV is an excellent skin prick inoculation vector. Both scratching/penetrating the skin surface and oral administration achieve desirable immune effects, resulting in animal protection. (ii) SPV demonstrates strict host specificity. Both deletion mutants and wtSPV are highly specific hosts. (iii) Used as a priming vaccine, SPV induces significantly increased cellular and humoural immune responses in pig models. Based on this study, gene deletion of the swinepox virus may generate additional innovative and efficient vaccine vector candidates.

## References

[1] Moss B (1996). Genetically engineered poxviruses for recombinant gene expression, vaccination, and safety. Proc Natl Acad Sci U S A.

[2] Paoletti E (1996). Applications of pox virus vectors to vaccination: an update. Proc Natl Acad Sci U S A.

[3] Lin H, Ma Z, Hou X, Chen L, Fan H (2017). Construction and immunogenicity of a recombinant swinepox virus expressing a multi-epitope peptide for porcine reproductive and respiratory syndrome virus. Sci Rep.

[4] Xu J, Huang D, Liu S, Lin H, Zhu H, Liu B, Chen W, Lu C (2013). Immune responses and protective efficacy of a recombinant swinepox virus co-expressing HA1 genes of H3N2 and H1N1 swine influenza virus in mice and pigs. Vet Microbiol.

[5] Xu J, Huang D, Liu S, Lin H, Zhu H, Liu B, Lu C (2012). Immune responses and protective efficacy of a recombinant swinepox virus expressing HA1 against swine H1N1 influenza virus in mice and pigs. Vaccine.

[6] Xu J, Huang D, Liu S, Lin H, Zhu H, Liu B, Lu C (2012). Immune responses and protection efficacy of a recombinant swinepox virus expressing HA1 against swine H3N2 influenza virus in mice and pigs. Virus Res.

[7] Huang D, Zhu H, Lin H, Xu J, Lu C (2012). First insights into the protective effects of a recombinant swinepox virus expressing truncated MRP of *Streptococcus suis* type 2 in mice. Berl Munch Tierarztl Wochenschr.

[8] Yuan X, Lin H, Fan H (2015). Efficacy and immunogenicity of recombinant swinepox virus expressing the A epitope of the TGEV S protein. Vaccine.

[9] Massung RF, Moyer RW (1991). The molecular biology of swinepox virus. I. A characterization of the viral DNA. Virology.

[10] Afonso CL, Tulman ER, Lu Z, Zsak L, Osorio FA, Balinsky C, Kutish GF, Rock DL (2002). The genome of swinepox virus. J Virol.

[11] Panicali D, Paoletti E (1982). Construction of poxviruses as cloning vectors: insertion of the thymidine kinase gene from herpes simplex virus into the DNA of infectious vaccinia virus. Proc Natl Acad Sci U S A.

[12] Shen X, Wong SB, Buck CB, Zhang J, Siliciano RF (2002). Direct priming and cross-priming contribute differentially to the induction of CD8+ CTL following exposure to vaccinia virus via different routes. J Immunol.

[13] Ito K, Shinohara N, Kato S (2003). DNA immunization via intramuscular and intradermal routes using a gene gun provides different magnitudes and durations on immune response. Mol Immunol.

[14] Taracha EL, Bishop R, Musoke AJ, Hill AV, Gilbert SC (2003). Heterologous priming-boosting immunization of cattle with *Mycobacterium tuberculosis* 85A induces antigen-specific T-cell responses. Infect Immun.

[15] Anderson RJ, Hannan CM, Gilbert SC, Laidlaw SM, Sheu EG, Korten S, Sinden R, Butcher GA, Skinner MA, Hill AV (2004). Enhanced CD8+ T cell immune responses and protection elicited against *Plasmodium berghei* malaria by prime boost immunization regimens using a novel attenuated fowlpox virus. J Immunol.

[16] Kim JJ, Nottingham LK, Sin JI, Tsai A, Morrison L, Oh J, Dang K, Hu Y, Kazahaya K, Bennett M, Dentchev T, Wilson DM, Chalian AA, Boyer JD, Agadjanyan MG, Weiner DB (1998). CD8 positive T cells influence antigen-specific immune responses through the expression of chemokines. J Clin Invest.

[17] Boyer JD, Kim J, Ugen K, Cohen AD, Ahn L, Schumann K, Lacy K, Bagarazzi ML, Javadian A, Ciccarelli RB, Ginsberg RS, MacGregor RR, Weiner DB (1999). HIV-1 DNA vaccines and chemokines. Vaccine.

[18] Xin KQ, Lu Y, Hamajima K, Fukushima J, Yang J, Inamura K, Okuda K (1999). Immunization of RANTES expression plasmid with a DNA vaccine enhances HIV-1-specific immunity. Clin Immunol.

[19] Kim JJ, Tsai A, Nottingham LK, Morrison L, Cunning DM, Oh J, Lee DJ, Dang K, Dentchev T, Chalian AA, Agadjanyan MG, Weiner DB (1999). Intracellular adhesion molecule-1 modulates beta-chemokines and directly costimulates T cells in vivo. J Clin Invest.

[20] Lu Y, Xin KQ, Hamajima K, Tsuji T, Aoki I, Yang J, Sasaki S, Fukushima J, Yoshimura T, Toda S, Okada E, Okuda K (1999). Macrophage inflammatory protein-1alpha (MIP-1α) expression plasmid enhances DNA vaccine-induced immune response against HIV-1. Clin Exp Immunol.

[21] Gherardi MM, Ramirez JC, Esteban M (2000). Interleukin-12 (IL-12) enhancement of the cellular immune response against human immunodeficiency virus type 1 env antigen in a DNA prime/vaccinia virus boost vaccine regimen is time and dose dependent: suppressive effects of IL-12 boost are mediated by nitric oxide. J Virol.

[22] Kim JJ, Yang JS, VanCott TC, Lee DJ, Manson KH, Wyand MS, Boyer JD, Ugen KE, Weiner DB (2000). Modulation of antigen-specific humoral responses in rhesus macaques by using cytokine cDNAs as DNA vaccine adjuvants. J Virol.

[23] Ahlers JD, Belyakov IM, Berzofsky JA (2003). Cytokine, chemokine, and costimulatory molecule modulation to enhance efficacy of HIV vaccines. Curr Mol Med.

[24] Moss B (2011). Smallpox vaccines: targets of protective immunity. Immunol Rev.

[25] Mayr A, Danner K (1978). Vaccination against pox diseases under immunosuppressive conditions. Dev Biol Stand.

[26] Stickl H, Hochstein-Mintzel V, Mayr A, Huber HC, Schafer H, Holzner A (1974). MVA vaccination against smallpox: clinical tests with an attenuated live vaccinia virus strain (MVA) (author’s transl). Dtsch Med Wochenschr.

[27] Antoine G, Scheiflinger F, Dorner F, Falkner FG (1998). The complete genomic sequence of the modified vaccinia Ankara strain: comparison with other orthopoxviruses. Virology.

[28] Meisinger-Henschel C, Schmidt M, Lukassen S, Linke B, Krause L, Konietzny S, Goesmann A, Howley P, Chaplin P, Suter M, Hausmann J (2007). Genomic sequence of chorioallantois vaccinia virus Ankara, the ancestor of modified vaccinia virus Ankara. J Gen Virol.

[29] Meyer H, Sutter G, Mayr A (1991). Mapping of deletions in the genome of the highly attenuated vaccinia virus MVA and their influence on virulence. J Gen Virol.

[30] Nakano E, Panicali D, Paoletti E (1982). Molecular genetics of vaccinia virus: demonstration of marker rescue. Proc Natl Acad Sci U S A.

[31] Prabhu N, Prabakaran M, Ho HT, Velumani S, Qiang J, Goutama M, Kwang J (2009). Monoclonal antibodies against the fusion peptide of hemagglutinin protect mice from lethal influenza A virus H5N1 infection. J Virol.

[32] Xie X, Lin Y, Pang M, Zhao Y, Kalhoro DH, Lu C, Liu Y (2015). Monoclonal antibody specific to HA2 glycopeptide protects mice from H3N2 influenza virus infection. Vet Res.

[33] Tian H, Wu J, Chen Y, Zhang K, Shang Y, Liu X (2012). Development of a SYBR green real-time PCR method for rapid detection of sheep pox virus. Virol J.

[34] Tang M, Harp JA, Wesley RD (2002). Recombinant adenovirus encoding the HA gene from swine H3N2 influenza virus partially protects mice from challenge with heterologous virus: A/HK/1/68 (H3N2). Arch Virol.

[35] Du Y, Li Y, He H, Qi J, Jiang W, Wang X, Tang B, Cao J, Jiang P (2008). Enhanced immunogenicity of multiple-epitopes of foot-and-mouth disease virus fused with porcine interferon alpha in mice and protective efficacy in guinea pigs and swine. J Virol Methods.

[36] Smellie RM, Keir HM, Davidson JN (1959). Studies on the biosynthesis of deoxyribonucleic acid by extracts of mammalian cells. I. Incorporation of tritium-labelled thymidine. Biochim Biophys Acta.

[37] Kit S (1985). Thymidine kinase. Microbiol Sci.

[38] Garcia MA, Gil J, Ventoso I, Guerra S, Domingo E, Rivas C, Esteban M (2006). Impact of protein kinase PKR in cell biology: from antiviral to antiproliferative action. Microbiol Mol Biol Rev.

[39] Hotamisligil GS (2010). Endoplasmic reticulum stress and the inflammatory basis of metabolic disease. Cell.

[40] Kulski JK, Shiina T, Anzai T, Kohara S, Inoko H (2002). Comparative genomic analysis of the MHC: the evolution of class I duplication blocks, diversity and complexity from shark to man. Immunol Rev.

